# The use of strong analgesics for prehospital pain management in children in the region of Southern Denmark: a register-based study

**DOI:** 10.1186/s13049-025-01339-w

**Published:** 2025-02-05

**Authors:** Josefine Tvede Colding-Jørgensen, Gina Maj Graven Brandstrup, Vibe Maria Laden Nielsen, Josefine Gradman, Line Anker Bang Thybo, Peter Martin Hansen, Daniel Wittrock, Stig Nikolaj Fasmer Blomberg, Helle Collatz Christensen, Søren Mikkelsen

**Affiliations:** 1 The Prehospital Research Unit, Region of Southern Denmark, Odense University Hospital, Odense, Denmark; 2 Centre for Prehospital and Emergency Research, Aalborg University and Aalborg University Hospital, Aalborg, Denmark; 3 Hans Christian Andersen Children’s Hospital, Odense University Hospital, Odense, Denmark; 4 Ambulance Syd, Odense, Denmark; 5 Prehospital Center, Region Zealand, Ringstedgade 61, 13th Floor, Næstved, 4700 Denmark; 6 Institute of Clinical Medicine, University of Copenhagen, Copenhagen, Denmark

**Keywords:** Prehospital analgesia, Prehospital paediatric patients, Prehospital opioids

## Abstract

**Background:**

Acute pain in the prehospital setting is frequent and prehospital pain management presents multiple challenges, especially in children. There is a lack of high-level evidence regarding prehospital pain management in the paediatric population worldwide. In Denmark, this lack of evidence particularly concerns the frequency of the prehospital use of strong analgesics. Guidelines are sparse but there is evidence that prehospital fentanyl may be administered up to 5 µg/kg.

**Method:**

This register-based study investigated the prehospital analgesic treatment in the population under 15 years from January 2017 to December 2022 in the Region of Southern Denmark. Data were extracted from electronic prehospital medical records. The analgesic treatment was characterised by the type of medication, dosage, administration method, and cause of ambulance dispatch. Lastly, response- and transport times were registered.

**Results:**

A total of 28,933 prehospital paediatric medical records were examined. In one in seventeen of all prehospital contacts with children, fentanyl, alfentanil, morphine and/or s-ketamine was administered. Three-quarters of the doses of strong analgesics were administered to patients older than 10 years. Fentanyl was the most frequently administered medication (96.4%). The median fentanyl-equipotent doses of opioids were 1.7 µg/kg adjusted according to standardised patient weight. In 63.4% of cases, the analgesic treatment was administered intravenously.

**Conclusion:**

The doses of opioids as administered by the EMS personnel seem safe as 97% of the doses were within the recommended range and even at the lower end of the recommended range. Although apparently safe, the utilisation of strong analgesics points to a risk of under-treating pain in children.

## Introduction

Patients in contact with the Emergency Medical Service (EMS) experience pain as a prominent symptom with a prevalence of 42–53% [1, 2]. The most common causes of moderate to severe pain in the prehospital setting involve external causes (e.g. injury), musculoskeletal diseases, and digestive system disorders [2, 3]. Effective analgesic treatment is a key outcome measure in prehospital care for children. The patients´ families regard it as a primary factor influencing their perception of the prehospital system’s management of the incident [4].

Adequate pain management is difficult [5], and inadequately treated pain can have negative effects on both physical and psychological function [5, 6]. Children are especially at risk of long-term negative consequences on pain response and pain tolerance [7]. The most common adverse effects from high doses of opioids include respiratory depression, sedation, bradycardia, nausea, and vomiting. Other clinical signs of overdose include miosis, muscle rigidity, constipation, and histamine release resulting in hypotension and urticaria [8].

Several studies show that pain in the prehospital setting may be inadequately treated due to multiple challenges: inaccurate pain assessment, insufficient knowledge or experience with administrating opioids and other analgesics, or concerns about adverse effects [3, 9–11].

Accurate pain assessment is complex, and several tools using behaviour, self-reporting, or physiological measures may be required to aid the clinician [12–15]. Self-reporting of pain is regarded as the most valid measure (e.g., visual analogue scales or numeric ranking scales [12, 15]). A valid self-reporting depends on sufficient language development and cognitive ability to rate pain. Therefore, accurate pain assessment is especially challenging in children [15, 16]. To mitigate this, specific tools to rate pain in children such as the FLACC (Face, Legs, Activity, Cry, and Consolability) assessment scale have been developed [17, 18]. Other factors influencing pain assessment in children include difficulty for the health care professional to separate anxiety and pain or the child being in an unfamiliar setting [19, 20]. A further obstacle for pain assessment could be the child’s expectations of a negative consequence from being in pain, and the child may underrate the actual pain for fear of receiving analgesics by injections [19, 20].

The Danish guidelines for the pharmacological treatment of pain include both opioid and non-opioid agents [21]. Fentanyl and s-ketamine are commonly used to treat severe pain in the EMS [2, 22, 23]. The Danish Pharmaceutical Information System recommends up to 4 µg/kg of fentanyl to children aged 2–11 years and up to 10 µg/kg when aged 12–14 years during general anaesthesia [24].

Only a few studies have addressed prehospital analgesia in children. In these studies, fentanyl has been administered in doses of 0.33–5.0 µg/kg [25]. The medication can be administered in several ways and intranasal administration of fentanyl has been preferred in the paediatric population [24, 26, 27]. There is a lack of high-level evidence on prehospital pain management, especially in children, who may be at greater risk of receiving inadequate analgesia [25, 28].

The overall aim of this retrospective, register-based study was to describe the prehospital administration of strong analgesics to the paediatric population (< 15 years) in the Region of Southern Denmark. The specific objectives were to:

1. Determine the incidence of children in contact with the EMS who received strong analgesic treatment defined as alfentanil, morphine, fentanyl, or s-ketamine.
2. Characterise the paediatric population receiving prehospital analgesic treatment in terms of age and sex, the selected criterion for ambulance dispatch, the ambulance response time, the time spent at the scene, and the transport time.
3. Describe the analgesic treatment in terms of the type of medication, administration route, and dosage.

## Methods

### Study setting and study population

The Region of Southern Denmark has a population of 1,238,406 citizens and covers 12,262 km^2^. The region is one of Denmark´s five health regions and is responsible for regional health care. The healthcare system is tax-funded and free at the point of contact all citizens. In all Danish health regions, the prehospital system is composed of a three-tiered system, with ambulances as the basic resource, a rapid response vehicle staffed with a paramedic as the second tier, and a rapid response unit (car or helicopter) staffed with a specialist in anaesthesiology as the third tier [29]. The level of the prehospital response is determined by dispatchers at the regional Emergency Medical Dispatch Centre according to the urgency (from an acute potentially life-threatening mission to advice/taxi/directing to other healthcare services, etc.) and the severity of a case. A decision-making tool that incorporates the patient’s complaint into one of 37 dispatch criteria, each representing a symptom or an injury (The Danish Index of Emergency Assistance), supports the decision concerning the prehospital response (tier and urgency) [30, 31]. When assigning a given mission a dispatch criterion, the Danish Index of Emergency Assistance indicates whether the dispatcher should dispatch the ambulance with lights and sirens (high-acuity mission) or without lights and sirens (low-acuity mission). The assigned dispatch criterion is registered in the prehospital medical record system. The prehospital care provider documents the treatment given in a nationwide electronic prehospital medical record which includes the prehospital findings, vital parameters, treatments administered, and patient characteristics (including the unique patient identifier, the Civil Personal Registration number (CPR-number)) [32, 33].

### Data sources

The national prehospital electronic patient medical record system has existed since 2015. The prehospital record includes information about the treating healthcare professionals, the clinical findings, the individual administrations of treatment, and timestamps for each EMS response [29]. The data are recorded by the attending EMS personnel. All emergency medical technicians and paramedics are authorised to administer fentanyl in doses up to 2 µg/kg [34]. In the Region of Southern Denmark, a prehospital physician is always assigned to paediatric patients requiring immediate prehospital care (i.e., ambulances dispatched with lights and sirens) as per regional guidelines. Further, the administration of fentanyl in patients weighing less than 25 kg requires the paramedic to consult a prehospital anesthesiologist over the telephone.

### Data collection

Data were extracted from the electronic prehospital records using the CPR number as a unique identifier [33]. The initial data extraction included all patients under the age of 15 years in contact with the Southern Denmark EMS between January 1, 2017 and December 31, 2022. From this cohort, we identified all paediatric patients receiving prehospital fentanyl, morphine, s-ketamine, or alfentanil during the study period and subjected them to further analysis. Intubated children were also included to understand the overall use of prehospital analgesic medication.

### Data storage and management

All data were pseudonymised. Following data management, data were exported to R (v 4.1.2, R Core Team, 2022, Vienna, Austria) for analysis. All person identifiable data were stored on encrypted servers hosted by the Region of Southern Denmark.

### Analyses

The analyses were divided into the following three parts to describe the study population and analgesic treatment:

The prevalence and the corresponding 95% confidence interval was determined by calculating the proportion of paediatric contacts receiving strong analgesic treatment out of all paediatric EMS contacts during the study period.

The study population receiving strong analgesic treatment was characterised by calculating percentages and the corresponding 95% confidence intervals for sex and age with age stratified into three categories. The age was also described by calculating the median and corresponding inter quartile ranges (IQR). The study population was further described with age as a continuous variable stratified by sex.

Lastly, the analgesic treatment was described to account for the possibility of one patient receiving more than one medication during one EMS encounter. Specifically this was done by aggregating the individual administrations for each contact according to the type of strong analgesic medication. The type of medication (alfentanil, fentanyl, morphine, and/or s-ketamine) was calculated as percentages of all analgesic treatments with the corresponding 95% confidence intervals. The administration routes were described for opioids and s-ketamine, respectively. All units were converted to µg. The doses of alfentanil and morphine were converted to equipotent doses of fentanyl: 1000 µg morphine as equal to 10 µg fentanyl and 1000 µg alfentanil as equal to 100 µg fentanyl. S-ketamine was not included in this or the following analyses due to its different mechanism of action. Equipotent doses of alfentanil and morphine, and the cumulated doses of fentanyl are reported as µg/kg. Patient weight is rarely, if at all, registered in the prehospital setting. Thus, for all patient cases an estimated weight was obtained from a standardised age-weight curve for Danish children [35]. The specific weight points were read by two authors individually (JTC-J and GMGB) and if the data points differed, a mean value was calculated. Median dosage and interquartile ranges (IQR) were calculated and stratified by age. Doses above 5 µg/kg were classified as above the maximum reported dose for prehospital analgesia [25].

Ambulance time spent at the scene and transport time was calculated as medians (IQR). These two time variables were combined to show the total time that each child was in EMS care.

Opioid administration related to time spent in EMS care for all cases was analysed by linear regression. Time spent in EMS care was calculated by adding time spent at the scene and transport time to hospital.

The criteria for ambulance dispatch as assigned by the dispatcher at the Emergency Medical Dispatch Centre according to the Danish Index for Emergency Care was reported as proportisons with 95% confidence intervals [31].

Intubated children were described or analysed individually in parts of the analysis, as these children likely received stronger analgesic treatment as part of the intubation.

### Approvals

This project was approved by the Judicial office of The Region of Southern Denmark (Ref. No. 23/34152). No further ethical approvals are necessary according to Danish law [36]. Data were stored on an encrypted server, and all data handling was carried out according to the Danish and European legislation concerning person-identifiable data [37, 38].

## Results

**Fig. 1:**
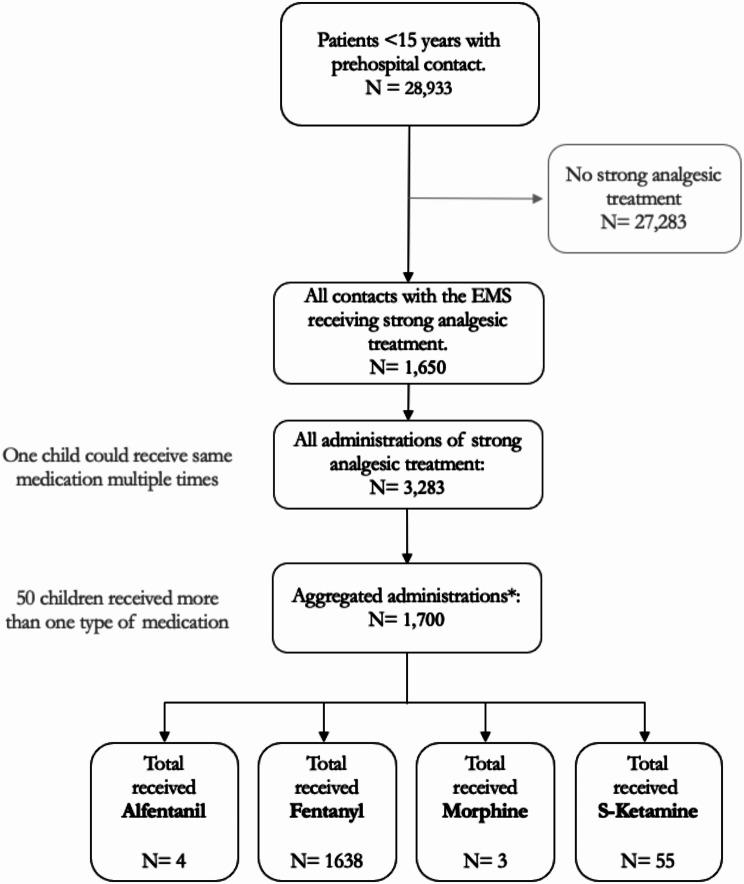
Flowchart visualising the data extraction process with exclusion criteria. * The 3,283 administrations were aggregated into 1,700 analgesic treatments in 1,650 children

The initial data extraction included 28,933 patients younger than 15 years having contact with the EMS between January 2017 and December 2022. Of the total population, 1,650 children (5.7% (95% CI: 4.6–6.8) received strong analgesic treatment (see Fig. 1). In 30 of the pediatric contacts (1.8%, 95% CI: 1.3–2.6) the analgesic treatment was given in conjunction with induction of anaesthesia and tracheal intubation.

**Fig. 2:**
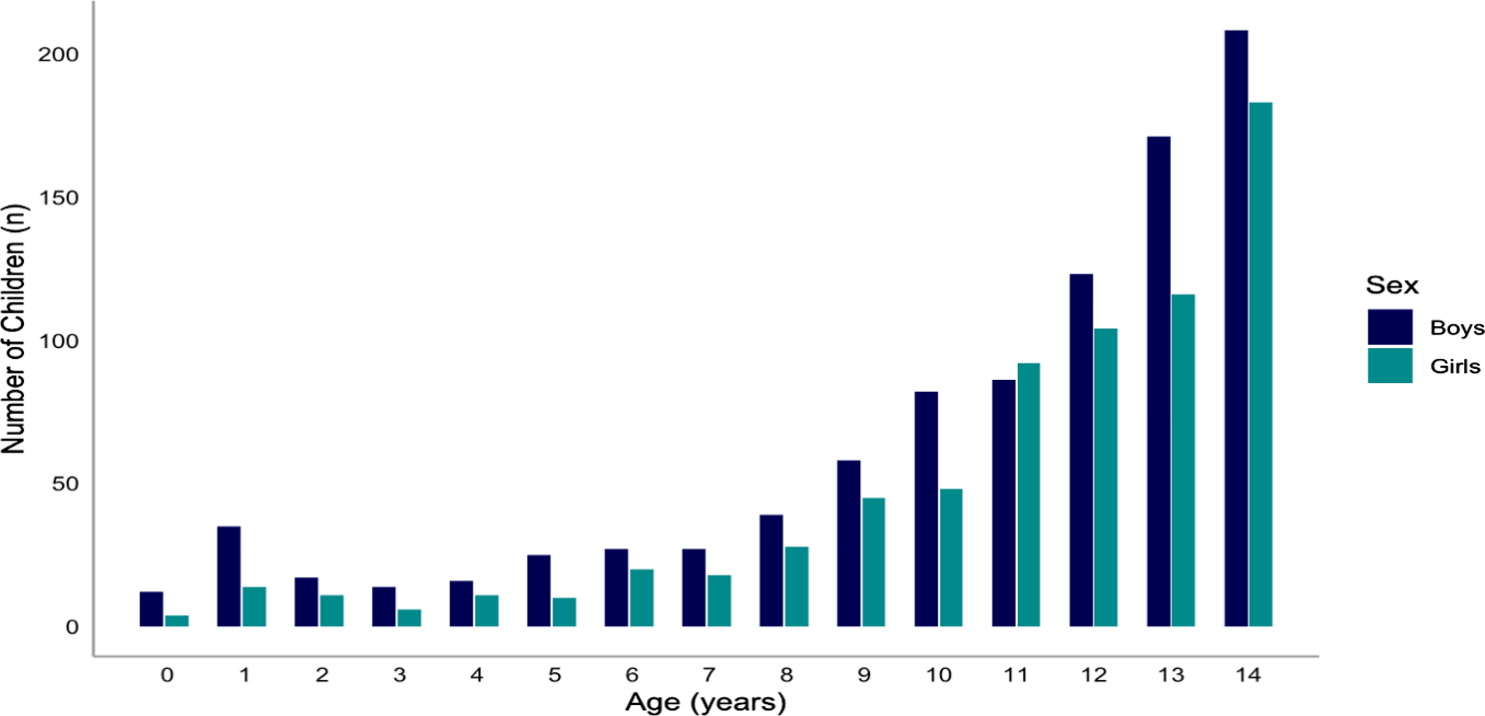
Age distribution of the study population stratified by sex, *N*=1,650

The study population consisted of 940 boys (57.0%) and 710 girls (43.0%). The median age was 12 years (IQR 9–13) The majority of the children (73.5%) were between 10 and 14 years old while 8.5% were below the age of 5 years (see Table 1). The distribution of age and sex is shown in Fig. 2.

**Table 1:** Characteristics of the paediatric population <15 years receiving prehospital strong analgesic treatment in the region of Southern Denmark (Jan. 2017– Dec. 2022) in terms of sex, age, +/- tracheal intubation, and type of received medication

	Total, *n* (%)
**Sex **(*N***= 1650)**	
Boys	940 (57.0)
Girls	710 (43.0)
**Age**, **years **(*N***= 1650)**	
< 5, n(%)	140 (8.5)
5– 9	297 (18.0)
10– 14	1213 (73.5)
**Tracheal intubation **(*N***=1650)**	
Intubated	30 (1.8)
Non-intubated	1620 (98.2)
**Type of received medication **(*N*=1700 administrations*)	
Fentanyl, n(%)	1638 (96.4)
S-Ketamine	55 (3.2)
Alfentanil	4 (0.2)
Morphine	3 (0.2)

*50 children received more than one type of analgesics

### Type of analgesic medication

The 1,650 children received a total of 3,283 individual administrations of analgesics. The 3,283 administrations were aggregated into 1,700 analgesic treatments as 50 children received more than one type of drug: 47 children received s-ketamine, two received alfentanil, and one received morphine in addition to fentanyl. In 55.5% of the cases (*n* = 943), the medication was administered in multiple doses. A minor proportion of the children, 3% (*n* = 52), received five administrations or more of the same medication.

Opioids were administered to 1,642 patients. The most frequently used analgesic treatment was fentanyl accounting for 96.4% of the 1,700 treatments (95% CI: 95.5–97.1). S-ketamine was used in 3.2% (95% CI: 2.5–4.2), and alfentanil and morphine were both administered to 0.2% of the patients (95% CI: alfentanil 0.09–0.6, morphine 0.06–0.5) (Table 1). All 30 intubated children received fentanyl, and seven children received both fentanyl and s-ketamine.

### Administration route

All three opioids were administered parenterally. 1,077 patients received fentanyl by intravenous route (63.4% (95% CI 61.0% − 65.6%). Twenty-one patients got fentanyl intramuscularly (1.2% (95% CI 0.1% − 1.9%). Sixteen patients received fentanyl by the intraosseous route (0.9% (95% CI 0.5% − 1.5%), while 586 had fentanyl administrated via nasal spray (34.5% (95% CI 32.2% − 36.8%). Similarly, the administration routes for s-ketamine were: seven intramuscular injections, two intraosseous administrations, 45 intravenous injections, and two doses administered via nasal spray. One child received s-ketamine by two different administration routes.

### Equipotent doses of opioids

The dose of the administered medication was recorded for all cases. In 28 of the 3,283 administrations, the total dose of fentanyl registered in the electronic patient records exceeded 15 *mg*. Considering the pharmacological profile of fentanyl, these cases were considered typing errors and the denomination *mg* was changed to µg.

A patient weight was not registered in the prehospital record system for any of the children and standardised weight was used in all cases. The median fentanyl equipotent dose of all 1,645 administrations of alfentanil, morphine or fentanyl was 1.7 µg/kg (IQR: 1.0–2.5 µg/kg). The distribution of equipotent doses according to age is shown in Table 2. The 30 intubated children received a median fentanyl equipotent dose of 3.6 µg/kg (IQR: 1.9–4.9 µg/kg) while the 1,615 children who were not intubated received a total median dose of 1.7 µg/kg (IQR: 1.0-2.4).

**Table 2:** Doses of fentanyl and equipotent doses of alfentanil and morphine in µg / standardised weight according to age

	Total, *n* (%)	95% CI
**Sex **(*N***= 1650)**		
Boys	940 (57.0)	4.6– 59.3
Girls	710 (43.0)	40.7– 45.4
**Age**, **years **(*N***= 1650)**		
< 5	140 (8.5)	7.2– 9.9
5– 9	297 (18.0)	16.2– 19.9
10– 14	1213 (73.5)	71.3– 75.6
**Tracheal intubation** (*N***=1650)**		
Intubated	30 (1.8)	1.3– 2.6
Non-intubated	1620 (98.2)	97.4– 98.7
**Type of received medication** (*N*= 1700 administrations*)		
Fentanyl, n(%)	1638 (96.4)	95.4– 97.1
S-Ketamine	55 (3.2)	2.5– 4.2
Alfentanil	4 (0.2)	0.1– 0.6
Morphine	3 (0.2)	0.1– 0.5

*50 children received more than one type of analgesics

Of all the 1,645 administrations of fentanyl, morphine, and alfentanil, 52 (3.2%) exceeded a total dose of 5 µg/kg (equipotent doses) with a median dose of 6.8 (IQR: 5.9–8.5 µg/kg) as shown in Table 2. Nine of these children were intubated and received a median equipotent dose of 7.1 µg/kg fentanyl/kg (IQR: 6.1–8.5 µg/kg). A total of nine children received more than 10 µg/kg. Two of these children were intubated.

### Time in prehospital care

For time in prehospital care, see Table 3.

**Table 3:** Ambulance time spent at the scene, transport time to hospital, total time in EMS care

	Total, *n*	Median (IQR), minutes	Missing, *n*
Time spent on scene	1644	20.2 (15.7-26.4)	6
Transport to hospital	1598	29.1 (22.2-38.3)	52
Total time in prehospital care	1598	50.4 (40.3-62.0)	52

### Opioid administration related to time in prehospital care

For each minute in prehospital care, the opioid administration increased 0,5% (*p* < 0.0001, R^2^ = 0.013).

### Dispatch codes determining the urgency of the ambulance

A Danish Index for Emergency Care dispatch code was assigned for 1,635 of the 1,650 contacts leaving 15 missing values [31]. One should bear in mind that dispatch codes concerning accidents and injuries to the extremities may overlap. However, only one dispatch code is assigned to every mission.

The 10 most frequent dispatch codes are presented in Table 4.

**Table 4:** The 10 most frequent ambulance dispatch criteria defined by index group in the Danish Index for Emergency Care (*n*=1650)

	Dispatch criterion	Total, *n* (%)*	95%CI
1	Accidents (not traffic-related)	676 (41.0)	38.6– 43.5
	(Danish index code 33)		
2	Extremities– wounds, fractures, minor injuries	436 (26.4)	24.4– 28.6
	(Danish index code 31)		
3	Transportation ordered by other health care provider.	150 (9.1)	7.8– 10.6
	(Danish index code 05)		
4	Traffic accident	84 (5.1)	4.1– 6.3
	(Danish index code 32)		
5	Abdominal pains	77 (4.7)	3.7– 5.8
	(Danish index code 24)		
6	Undefined problem	68 (4.1)	3.3– 5.2
	(Danish index code 06)		
7	Burns - electric cause	67 (4.1)	3.2– 5.1
	(Danish index code 09)		
8	“Unspecified Sick child”	24 (1.5)	0.9– 0.2
	(Danish index code 30)		
9	Convulsions	15 (0.9)	0.6– 1.5
	(Danish index code 23)		
10	Respiratory problems	10 (0.6)	0.3– 1.1
	(Danish index code 28)		

* 15 missing values

Fentanyl was the most frequently used analgesic in all individual causes of ambulance dispatches except for dispatches to patients who were assigned the ’Psychiatry/suicide’ index code in the Danish Index for Emergency Care which had equal proportions of treatment with fentanyl and s-ketamine [31].

## Discussion

Only 5.7% of the prehospital patient population below the age of 15 years of age received strong analgesics. Three-quarters of these patients were above 10 years of age. The main analgesic drug used was fentanyl with an estimated median dose of 1.7 µg/kg. The vast majority of the analgesic treatments were administered intravenously and to patients who were assigned dispatch codes describing traumatic injury. Only a very small proportion of patients were less than one year old and they received very small doses of fentanyl.

### Other studies

We found that the majority of analgesic administrations occurred in relation to trauma. This is in line with Whitley et al. who found that pain caused by trauma tended to be treated sooner than non-traumatic pain [9]. More boys than girls were given a prehospital analgetic. One explanation could be that boys are more often transported in ambulances than girls [39]. Another possible explanation could be that boys may tend to become more severely injured than girls [40].

Pain is experienced differently depending on gender. A study by Fillingim et al. thus reported that girls tend to be more sensitive to pain than boys [41]. Whitley et al. found that being a boy was a predictor of better pain management and discuss that a reason might be an unconscious gender bias or possibly information bias in terms of how boys report pain relief (trying to act tough and thus reporting greater pain relief) [9].

Younger children with fractures or burns are found less often to be given analgesics prehospitally than older children [42]. Our findings that older children constitute the majority of the population receiving strong analgesics support this finding. In contrast, however, Whitley et al. found younger age as a predictor for better pain management although one limitation of that study reportedly was the inaccuracy in the use of pain assessment scales among small children, potentially overestimating the effect of analgesic treatment [9, 27].

In our study the majority of opioid administrations were intravenous. This deviates somewhat from what is reported or recommended in other studies where the application of analgesics on the nasal mucosa has been recommended as intranasal fentanyl has been shown to have pharmacokinetic and pharmacodynamics dynamics that are desirable for the management of acute pain [26, 27, 43, 44]. A possible explanation may be the combination of two factors: Danish paramedics are authorised to perform intravenous cannulation including in children, and the majority of the children in our cohort are in the age group 10–14 years. We speculate that there a lower threshold for inserting intravenous access in an older child. This study was carried out in one of Denmark’s five regions covering 1.2 million inhabitants of both urban and rural populations. The study population is probably representative of most of Denmark, making the external validity acceptable. The internal validity is supported by our study relying on data that were collected from the electronic prehospital record system. This system allows both online registration and post hoc registration and is considered an acceptable method of registration [45, 46].

Most of the doses of opioids administered to the children are within the recommended range [24]. In the cases where the total dose of fentanyl (or equipotent analgesics) exceeded the maximum recommended dose of 5 µg/kg, the median dose was 6.8 µg/kg (IQR: 5.9–8.5). The intubated children received a higher median dose than children not intubated. Thus, any considerations concerning respiratory depression would be set aside by the instituted ventilator therapy. A small proportion of the non-intubated children received a higher total dose of fentanyl than 10 µg/kg. These higher doses of fentanyl correlated with longer time spent in ambulance care.

The time spent at the scene and the transport time to the hospital add up to a median of 50 min in ambulance care. Considering the duration of time in prehospital care and the fact that most doses were given through multiple administrations, even the slightly higher total doses may still be safe and relevant. Taking a median time of 50 min in ambulance care into consideration, it is, however, somewhat surprising that only approximately half of the children were treated with multiple administrations. As the majority of the population had trauma-induced pain, our findings may reflect an approach where an initial treatment with fentanyl was administered until any pain-provoking procedures such as reduction of fractures were completed [27].

### Limitations of the study

The use of the electronic prehospital record system presents some limitations. First of all, our data do not allow for any analyses concerning over- or under-utilisation of strong analgesics as the data from the prehospital patient medical records used in this study did not include free text fields of the prehospital medical record. Thus, written observations of clinical signs and the explicated indications for treatment as well as written recordings of side effects were not included in the data. This is a limitation. Second, the register-based data did not include all data for every paediatric contact. In particular, pain scores were almost totally absent in the medical records. This, as well as some missing cases and omission of factors such as patient weight possibly impacting the accuracy of our results. We have sought to compensate for the lack of data on patient weight by using an age-standardised weight curve. This approach requires that no patients be over- or underweight, and our approach may have resulted in limited data accuracy. However, in a prehospital setting, the exact weight is often not available at the time of administration and the titration of doses must then be based on an estimation of the patient’s weight. Other studies show that using age-based weight assumption is not a very accurate measure and that it can lead to dosing errors [47–49]. Considering the large size of our population, we consider that the use of a standardised weight may be a feasible measure in our study. Thirdly, misregistrations can have impacted the results. The most obvious erroneously registered doses and units were corrected (28 doses recorded as 15 mg fentanyl were changed to 15 µg). Less obvious misregistrations might have gone undiscovered potentially impacting the accuracy of the results. Finally, multiple administrations might have been registered as one combined administration if registered post hoc. Therefore, there is a possibility that the number of individual administrations of opioids registered is inaccurate. Our finding that longer time in prehospital care is associated with increased cumulated doses of opioids is hampered by the very low R^2^ value. A low R^2^ value of 0.013 should be interpreted in a way that time in prehospital care in itself is not the sole factor determining cumulated opioid dose.

## Conclusion

Only a minor proportion of children (one child in 17) in contact with the EMS received analgesic treatment in the Region of Southern Denmark between 2017 and 2022. Fentanyl was the most used strong analgesic treatment and was most frequently administered intravenously and in relation to accidents. Although apparently safe, the utilisation of strong analgesics points to be a risk of under-treating pain in children.

## Data Availability

Data availability: Data are available in anonymised form from the corresponding author on reasonable request.

## Abbreviations

CI: Confidence Interval
CPR number: Civil Personal Register number
EMS: Emergency Medical System
IQR: Interquartile ranges

## References

[1] Galinski M, Ruscev M, Gonzalez G, Kavas J, Ameur L, Biens D, et al. Prevalence and management of acute pain in prehospital emergency medicine. Prehosp Emerg Care. 2010;14(3):334–9. 20507221 10.3109/10903121003760218

[2] McLean SA, Maio RF, Domeier RM. The epidemiology of pain in the prehospital setting. Prehosp Emerg Care. 2002;6(4):402–5. 12385606 10.1080/10903120290938021

[3] Friesgaard KD, Riddervold IS, Kirkegaard H, Christensen EF, Nikolajsen L. Acute pain in the prehospital setting: a register-based study of 41.241 patients. Scand J Trauma Resusc Emerg Med. 2018;26(1):53. 29970130 10.1186/s13049-018-0521-2 PMC6029421

[4] Pagnamenta R, Benger JR. Factors influencing parent satisfaction in a children’s emergency department: prospective questionnaire-based study. Emerg Med J. 2008;25(7):417–9. 18573954 10.1136/emj.2007.050005

[5] Sinatra R. Causes and consequences of inadequate management of Acute Pain. Pain Med. 2010;11(12):1859–71. 21040438 10.1111/j.1526-4637.2010.00983.x

[6] Schug SA, Palmer GM, Scott DA, Halliwell R, Trinca J. Acute Pain Management: scientific evidence, fourth edition, 2015. Med J Aust. 2016;204(8):315–7. 27125806 10.5694/mja16.00133

[7] Young KD. Pediatric procedural pain. Ann Emerg Med. 2005;45(2):160–71. 15671974 10.1016/j.annemergmed.2004.09.019

[8] Zöllner C, Stein C, Opioids. Handb Exp Pharmacol. 2007;177:31–63.10.1007/978-3-540-33823-9_2 10.1007/978-3-540-33823-9_2 17087119

[9] Whitley GA, Hemingway P, Law GR, Jones AW, Curtis F, Siriwardena AN. The predictors, barriers and facilitators to effective management of acute pain in children by emergency medical services: a systematic mixed studies review. J Child Health Care. 2021;25(3):481–503. 32845710 10.1177/1367493520949427 PMC8422593

[10] Berben SA, Meijs TH, van Grunsven PM, Schoonhoven L, van Achterberg T. Facilitators and barriers in pain management for trauma patients in the chain of emergency care. Injury. 2012;43(9):1397–402. 21371708 10.1016/j.injury.2011.01.029

[11] Whitley DE, Li T, Jones CMC, Cushman JT, Williams DM, Shah MN. An Assessment of newly identified barriers to and enablers for Prehospital Pediatric Pain Management. Pediatr Emerg Care. 2017;33(6):381–7. 26414634 10.1097/PEC.0000000000000514

[12] Jennings PA, Cameron P, Bernard S. Measuring acute pain in the prehospital setting. Emerg Med J. 2009;26(8):552–5. 19625547 10.1136/emj.2008.062539

[13] Jensen MP, Karoly P, Braver S. The measurement of clinical pain intensity: a comparison of six methods. Pain. 1986;27(1):117–26. 3785962 10.1016/0304-3959(86)90228-9

[14] Schwerin DL, Mohney S, EMS Pain Assessment And Management Treasure Island (FL). StatPearls Publishing; 2024. Jan-; 2023. Accessed 20 November 2024 at: https://www.ncbi.nlm.nih.gov/books/NBK554543/32119430

[15] Krusenstjerna-Hafstrøm MN, Jensen CS, Kirkegaard H, Galili SF. Acute pain treatment of children in the Danish emergency departments. Dan Med J. 2023,70(3). 36896722

[16] Beltramini A, Milojevic K, Pateron D. Pain Assessment in newborns, infants, and children. Pediatr Ann. 2017;46(10):e387–95. 29019634 10.3928/19382359-20170921-03

[17] Manworren RC, Hynan LS. Clinical validation of FLACC: preverbal patient pain scale. Pediatr Nurs. 2003;29(2):140–6. 12723828

[18] Willis MH, Merkel SI, Voepel-Lewis T, Malviya S. FLACC behavioral Pain Assessment Scale: a comparison with the child’s self-report. Pediatr Nurs. 2003;29(3):195–8. 12836995

[19] Drendel AL, Kelly BT, Ali S. Pain assessment for children: overcoming challenges and optimizing care. Pediatr Emerg Care. 2011;27(8):773–81. 21822093 10.1097/PEC.0b013e31822877f7

[20] Whitley GA, Hemingway P, Law GR, Siriwardena AN. Improving ambulance care for children suffering acute pain: a qualitative interview study. BMC Emerg Med. 2022;22(1):96. 35659188 10.1186/s12873-022-00648-y PMC9164349

[21] Yousefifard M, Askarian-Amiri S, Madani Neishaboori A, Sadeghi M, Saberian P, Baratloo A. Pre-hospital pain management; a systematic review of proposed guidelines. Arch Acad Emerg Med. 2019;7(1):e55. 31875209 PMC6905420

[22] Kiavialaitis GE, Müller S, Braun J, Rössler J, Spahn DR, Stein P, et al. Clinical practice of pre-hospital analgesia: an observational study of 20,978 missions in Switzerland. Am J Emerg Med. 2020;38(11):2318–23. 31785972 10.1016/j.ajem.2019.10.033

[23] Garrick JF, Kidane S, Pointer JE, Sugiyama W, Van Luen C, Clark R. Analysis of the paramedic administration of fentanyl. J Opioid Manag. 2011;7(3):229–34. 21823553

[24] Fentanyl B. Braun Medicin.dk - Professionel. Accessed 20 November 2024 at: https://pro.medicin.dk/Medicin/Praeparater/4608

[25] Cohen N, Cohen DM, Barbi E, Shavit I. Analgesia and Sedation of Pediatric patients with Major Trauma in Pre-hospital and Emergency Department Settings-A Narrative Review. J Clin Med. 2023,12(16). 10.3390/jcm12165260 PMC10455791 37629302

[26] Borland ML, Jacobs I, Rogers IR. Options in prehospital analgesia. Emerg Med (Fremantle). 2002;14(1):77–84. 11993839 10.1046/j.1442-2026.2002.00288.x

[27] Lindbeck G, Shah MI, Braithwaite S, Powell JR, Panchal AR, Browne LR, et al. Evidence-based guidelines for Prehospital Pain Management: recommendations. Prehosp Emerg Care. 2023;27(2):144–53. 34928760 10.1080/10903127.2021.2018073

[28] Rupp T, Delaney KA. Inadequate analgesia in emergency medicine. Ann Emerg Med. 2004;43(4):494–503. 15039693 10.1016/j.annemergmed.2003.11.019

[29] Mikkelsen S, Lassen AT. The Danish prehospital system. Eur J Emerg Med. 2020;27(6):394–5. 33105290 10.1097/MEJ.0000000000000774

[30] Andersen MS, Johnsen SP, Sørensen JN, Jepsen SB, Hansen JB, Christensen EF. Implementing a nationwide criteria-based emergency medical dispatch system: a register-based follow-up study. Scand J Trauma Resusc Emerg Med. 2013;21:53. 23835246 10.1186/1757-7241-21-53 PMC3708811

[31] Danske Regioner. Dansk Indeks for Akuthjælp 1.10. 2022. Accessed 20 November 2024 at: https://dpv.rn.dk/-/media/Hospitaler/Den-Praehospitale-Virksomhed/Dansk-Indeks-1-8-Landsudgaven.ashx?la=da

[32] Lindskou TA, Mikkelsen S, Christensen EF, Hansen PA, Jørgensen G, Hendriksen OM, et al. The Danish prehospital emergency healthcare system and research possibilities. Scand J Trauma Resusc Emerg Med. 2019;27(1):100. 31684982 10.1186/s13049-019-0676-5 PMC6829955

[33] Schmidt M, Pedersen L, Sørensen HT. The Danish Civil Registration System as a tool in epidemiology. Eur J Epidemiol. 2014;29(8):541–9. 24965263 10.1007/s10654-014-9930-3

[34] Friesgaard KD, Kirkegaard H, Rasmussen CH, Giebner M, Christensen EF, Nikolajsen L. Prehospital intravenous fentanyl administered by ambulance personnel: a cluster-randomised comparison of two treatment protocols. Scand J Trauma Resusc Emerg Med. 2019;27(1):11. 30732618 10.1186/s13049-019-0588-4 PMC6367789

[35] Tinggaard J, Aksglaede L, Sørensen K, Mouritsen A, Wohlfahrt-Veje C, Hagen CP, et al. The 2014 Danish references from birth to 20 years for height, weight and body mass index. Acta Paediatr. 2014;103(2):214–24. 24127859 10.1111/apa.12468

[36] Thygesen LC, Daasnes C, Thaulow I, Brønnum-Hansen H. Introduction to Danish (nationwide) registers on health and social issues: structure, access, legislation, and archiving. Scand J Public Health. 2011;39(7 Suppl):12–6. 21898916 10.1177/1403494811399956

[37] Regulation (EU) 2016/679 of the European Parliament and of the Council of 27 April 2016 on the protection of natural persons with regard to the processing of personal data and on the free movement of such data, and repealing Directive 95/46/EC (General Data Protection Regulation) (Text with EEA relevance). p 1. Accessed November 20. 2024 at: http://data.europa.eu/eli/reg/2016/679/oj

[38] The Ministry of Justice. Law on General Data Protection, supplemental to Regulation (EU) 2016/679 of the European Parliament and of the Council of 27 April 2016 on the protection of natural persons with regard to the processing of personal data and on the free movement of such data, and repealing Directive 95/46/EC (General Data Protection Regulation), LBK nr 289 af 08/023/2024. Accessed 20 November 2024 at: https://www.retsinformation.dk/eli/lta/2024/289

[39] Nielsen VML, Søvsø MB, Skals RG, Bender L, Corfield AR, Lossius HM, et al. Mortality after paediatric emergency calls for patients with or without pre-existing comorbidity: a nationwide population based cohort study. Scand J Trauma Resusc Emerg Med. 2024;32(1):48. 38807153 10.1186/s13049-024-01212-2 PMC11134704

[40] Rosen BN, Petersen L. Gender differences in children´s outdoor play injuries: a review and an integration. Clin Psychol Rev. 1990;10(2):187–205.

[41] Fillingim RB, King CD, Ribeiro-Dasilva MC, Rahim-Williams B, Riley JL 3. Sex, gender, and pain: a review of recent clinical and experimental findings. J Pain. 2009;10(5):447–85. 10.1016/j.jpain.2008.12.001 PMC2677686 19411059

[42] Watkins N. Paediatric prehospital analgesia in Auckland. Emerg Med Australas. 2006;18(1):51–6. 16454775 10.1111/j.1742-6723.2006.00808.x

[43] Prescott MG, Iakovleva E, Simpson MR, Pedersen SA, Munblit D, Vallersnes OM, Austad B. Intranasal analgesia for acute moderate to severe pain in children - a systematic review and meta-analysis. BMC Pediatr. 2023;23(1):405. 10.1186/s12887-023-04203-x. 37596559 10.1186/s12887-023-04203-x PMC10436645

[44] Foster D, Upton R, Christrup L, Popper L. Pharmacokinetics and pharmacodynamics of intranasal versus intravenous fentanyl in patients with pain after oral surgery. Ann Pharmacother. 2008;42(10):1380–7. 10.1345/aph.1L168. 18728103 10.1345/aph.1L168

[45] Kjær J, Milling L, Wittrock D, Nielsen LB, Mikkelsen S. The data quality and applicability of a Danish prehospital electronic health record: a mixed-methods study. PLoS ONE. 2023;18(10):e0293577. 37883522 10.1371/journal.pone.0293577 PMC10602337

[46] Poulsen NR, Kløjgård TA, Lübcke K, Lindskou TA, Søvsø MB, Christensen EF. Completeness in the recording of vital signs in ambulances increases over time. Dan Med J. 2020,67(2). 32053487

[47] Wells M, Henry B, Goldstein L. Weight estimation for drug dose calculations in the Prehospital setting - a systematic review. Prehosp Disaster Med. 2023;38(4):471–84. 37439214 10.1017/S1049023X23006027 PMC10445115

[48] Selbst SM, Fein JA, Osterhoudt K, Ho W. Medication errors in a pediatric emergency department. Pediatr Emerg Care. 1999;15(1):1–4. 10069301 10.1097/00006565-199902000-00001

[49] Wells M, Goldstein LN, Bentley A. The accuracy of emergency weight estimation systems in children-a systematic review and meta-analysis. Int J Emerg Med. 2017;10(1):29. 28936627 10.1186/s12245-017-0156-5 PMC5608658

